# Characterization of *Hottentotta judaicus* Scorpion Venom: Toxic Effects and Neurobehavioral Modulation in Insect Models

**DOI:** 10.3390/toxins17110546

**Published:** 2025-11-03

**Authors:** Rim Wehbe, Aline Karaki, Zeina Dassouki, Mohamad Rima, Adolfo Borges, Rabih Roufayel, Christian Legros, Ziad Fajloun, Zakaria Kambris

**Affiliations:** 1 Biology Department, Faculty of Arts and Sciences, American University of Beirut, Beirut 1107 2020, Lebanon; rgw00@mail.aub.edu (R.W.); aak108@mail.aub.edu (A.K.); 2 Department of Medical Laboratory Sciences, Faculty of Health Sciences, University of Balamand, Tripoli P.O. Box 100, Lebanon; zeina.dassouki@balamand.edu.lb; 3 Department of Biological Sciences, Lebanese American University, Byblos P.O. Box 36, Lebanon; mohamad.rima@lau.edu.lb; 4 Laboratorio de Biologia Molecular de Toxinas y Receptores, Instituto de Medicina Experimental, Facultad de Medicina, Universidad Central de Venezuela, Caracas 1050, Venezuela; borges.adolfo@gmail.com; 5 Centro para el Desarrollo de la Investigación Científica (CEDIC), Asunción 1255, Paraguay; 6 College of Engineering and Technology, American University of the Middle East, Egaila 54200, Kuwait; rabih.roufayel@aum.edu.kw; 7 Univ. Angers, INSERM, CNRS, MITOVASC, Equipe CarME, SFR ICAT, 49000 Angers, France; christian.legros@univ-angers.fr; 8 Department of Cell Culture, Laboratory of Applied Biotechnology (LBA3B), Azm Center for Research in Biotechnology and Its Applications, EDST, Lebanese University, Tripoli 1300, Lebanon; 9 Faculty of Sciences 3, Lebanese University, Michel Slayman Tripoli Campus, Ras Maska 1352, Lebanon

**Keywords:** scorpion venom, toxins, *Hottentotta judaicus*, cytotoxicity, *Drosophila melanogaster*, mosquitoes, insecticidal effects, locomotor activity

## Abstract

Scorpion venom is a rich source of diverse bioactive molecules with medicinal importance. While the venoms of many Buthidae scorpions have been extensively studied for their toxicity and therapeutic potential, *Hottentotta judaicus* scorpion venom (HjSV) remains poorly explored. In this study, using LC-ESI-MS, we show that HjSV has a complex composition. We find that HjSV has no significant cytotoxic effects on three human cancer cell lines, even at concentrations of up to 1000 µg/mL. However, it exerts a dose-dependent insecticidal effect against *Drosophila melanogaster*, a well-established genetic model organism, and two medically relevant mosquito species, *Aedes albopictus* and *Culex pipiens*. These findings highlight the venom’s selective activity and reveal a species-dependent susceptibility in insects, with mosquitoes being more sensitive than *Drosophila*. Furthermore, we show that at sub-lethal doses, HjSV alters *D. melanogaster* behavioral patterns, significantly reducing locomotor activity and increasing sleep duration. Altogether, our results provide new insights into the dual role of HjSV as both an insecticidal agent and behavioral modulator, shedding light on its ecological function in prey subduing and its potential application in pest control strategies.

## 1. Introduction

Scorpions are one of the most ancient groups of arachnids that have successfully occupied a vast range of ecological niches [1]. They play important roles in the ecosystem, contributing to both biodiversity and ecological balance. Among the nearly 2000 described scorpion species worldwide, *Hottentotta judaicus* (*H. judaicus*) belongs to the family Buthidae, which represents the largest and most medically important group of scorpions [2,3]. *H. judaicus*, commonly known as the black Judaicus scorpion, is broadly spread across the Levant region of the Middle East, including Lebanon [4]. *H. judaicus* is a nocturnal predator that feeds primarily on insects such as cockroaches, crickets and beetles [5]. A study investigating the feeding preference of *H. tumulus* (a closely related species to *H. judaicus*) found that although they feed mainly on crickets, they consumed the flies *Musca domestica* when they were not given the choice [6]. This opportunism is an important aspect of scorpions’ feeding ecology, allowing them to exploit a diversity of prey in their environment. Scorpions inject their venom via the stinger to rapidly immobilize or kill their targets [7]. It is worth noting that unlike other members of the Buthidae family, *H. judaicus* is not considered dangerous to humans, and its sting generally causes only mild symptoms [8]. Mild scorpion envenomation is typically characterized by local symptoms at the sting site, such as pain, redness, and swelling, which are reported in more than half of the cases [9,10].

Scorpion venom is a rich mixture of several components including free amino acids, peptides and proteins. These proteins are mainly enzymes that the scorpion uses for defense and capture mechanisms [11]. Typically, the venom of Buthidae scorpions is mainly enriched with neurotoxic peptides (alpha and beta toxins) that disrupt ion channels [12]. These neurotoxins (also called long-chain toxins) can act on voltage-gated sodium channels (Nav) in insects, mammals, or both, depending on their structural specificity and target channel [13]. The toxins targeting insects are referred to as insectotoxins [14]. Based on their physiological effect on the host’s nervous system, the insectotoxins can act as excitatory toxins where they shift the activation voltage of the Na channels to negative values resulting in an uncontrollable insect muscle contraction. Alternatively, the insectotoxins can act as depressant neurotoxins where they slow down or block the inactivation of the Na channels resulting in an immobilized insect [15]. In addition to long-chain toxins, short-chain toxins present in the Buthidae scorpion venom exert their effects by primarily blocking voltage-gated potassium ion channels (Kv) [16]. Surprisingly, most of toxin transcripts detected in the resting venom gland of *H. judaicus* were potassium channel toxins, which is atypical in Buthidae venoms [17]. To date, three insectotoxins have been identified from *H. judaicus* venom: BjαIT (from its previous genus name *Buthotus judaicus;* a depressant α toxin), BjaIT2 (a depressant β toxin) and Bj-xtrIT (an excitatory β toxin) [14,18,19].

Several studies investigating the therapeutic effects of scorpion venom focused on chlorotoxin, a small peptide derived from *Leiurus quinquestriatus*. This toxin is one of the best-known scorpion-derived peptides in cancer therapy research, with applications ranging from tumor imaging to targeted drug delivery for gliomas (primary brain tumors) [20,21]. Some studies also explored the anti-microbial effect of scorpion venom where they found that the venom contains anti-microbial peptides (AMPs) which are small cationic molecules active against a wide range of both negative and positive bacteria. Other scorpion-derived AMPs also exhibited anti-fungal and anti-parasitical activities [22,23,24,25].

Despite the existing literature on scorpion venoms, the bioactivities of HjSV in particular remain poorly investigated. Early-stage research tested the insecticidal activity of HjSV toxins against cockroaches and blowfly larvae and showed that the venom’s toxins are potent at low doses [18,19]. A recent study also showed that HjSV modulates nociceptive response and reduces inflammatory cytokine levels in an experimental hyperalgesia mouse model, suggesting potential analgesic and anti-inflammatory properties [26].

In this study, we wanted to further characterize HjSV molecular profile and physiological effects on both mammalian cells and insects. For this, we analyzed HjSV using Liquid Chromatography Electrospray Ionization Mass Spectrometry (LC-ESI-MS). In addition, we assessed the cytotoxicity of HjSV on three different cancerous cell lines. More importantly, we tested HjSV insecticidal and behavioral effects—locomotor activity and sleep pattern—on the well-established model *Drosophila melanogaster* [27,28]. We also extended the analysis of HjSV effects to the mosquitoes *Aedes albopictus* and *Culex pipiens*, which are insects of medical importance given that they are one of the deadliest vectors transmitting infectious diseases to humans [29,30,31]. Although mosquitoes and *Drosophila* are not natural food targets for adult *H. judaicus*, they both can serve as useful paradigms to study venom bioactivity. Altogether, our results provide new insights into the bioactivities of the poorly studied *HjSV*, reinforcing its ecological role as an arthropod predator and its potential to be used as a future source of selective biopesticides.

## 2. Results

### 2.1. LC-ESI-MS Analysis of HjSV

**Figure 1:**
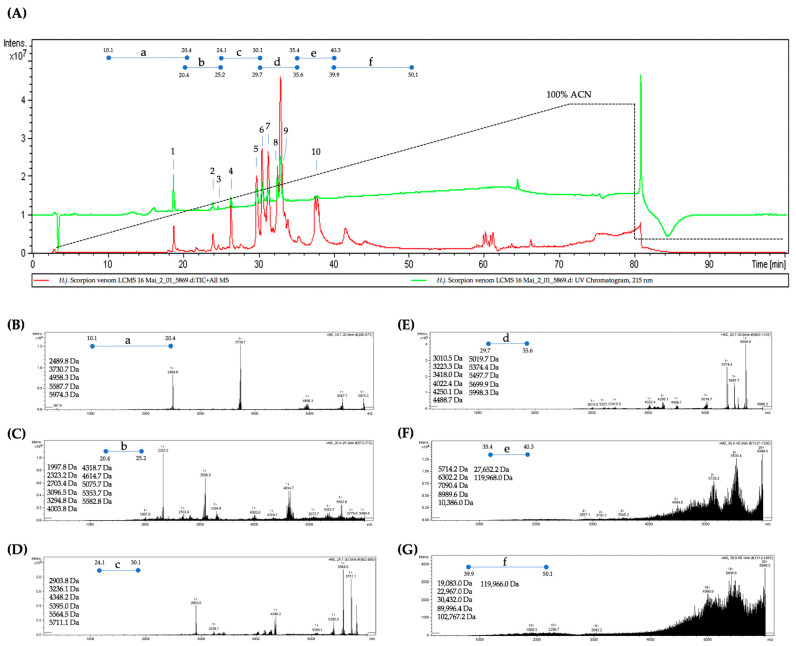
LC-ESI-MS analysis of HjSV reveals its complex composition. Analytical reverse-phase C18 HPLC profile of HjSV showing ten main fractions eluted at different retention times (green line) (**A**). The total ion chromatogram (TIC) corresponds to the ESI–MS mass spectrum of the eluted compounds (red line). The dotted line indicates the increase in acetonitrile (ACN) percentage during the elution gradient (0 to 100% ACN over 60 min). The ESI mass spectrum highlights the distribution of detected masses, particularly during the elution of the main LC-separated fractions. For improved resolution and interpretation of the detected masses, the LC–ESI–MS profile was divided into six segments (a, b, c, d, e, and f). Each segment was analyzed individually to identify the masses of the compounds present in the corresponding fractions. Panels (**B**–**G**) show the mass spectra of segments “a” to “f” of the LC–ESI–MS profile, covering the following intervals: (a) 10.1–20.4 min, (b) 20.4–25.2 min, (c) 25.1–30.1 min, (d) 29.7–30.6 min, (e) 35.4–40.3 min, and (f) 39.9–50.1 min. The main detected masses in each segment are indicated in Daltons (Da).

LC-ESI-MS analysis of HjSV provided a detailed profile of its complex protein and peptide composition, consistent with the recent comprehensive proteomic characterization of this venom [32]. The venom was subjected to high-performance reverse-phase liquid chromatography (RP-HPLC), revealing ten major peaks characterized by distinct retention times. The coupling of HPLC to electrospray ionization mass spectrometry (ESI-MS) enabled double detection of eluted constituents. This approach provided both a UV chromatogram and a mass spectrum of the venom (Figure 1A). This setup enabled an effective separation of the venom components based on their hydrophobicity and was complemented by a high-precision mass detection and characterization of the different constituents including low-abundance proteins and peptides. Indeed, we observed a wide range of molecules present in HjSV, with molecular masses ranging from 1997.8 Daltons (Da) to 119,966.0 Da. For better visualization and allocation of the molecular masses in the different RP-HPLC peaks or fractions, we segmented the LC–ESI–MS profile of the venom into six sections (a, b, c, d, e and f) as shown in Figure 1A. Each segment was then analyzed separately to identify the masses of the compounds present in the corresponding fractions. The mass spectra presented in Figure 1B–E (which correspond to segments a through d) show relatively low masses ranging between 2000 Da and 5000–6000 Da. The last two spectra in Figure 1F,G (which correspond to segments e and f) reveal the presence of compounds with very high molecular masses due to their hydrophobic features.

### 2.2. HjSV Exerts No Significant Cytotoxic Effect on U87, HT29 D4 and HTC116 Cancer Cells

**Figure 2:**
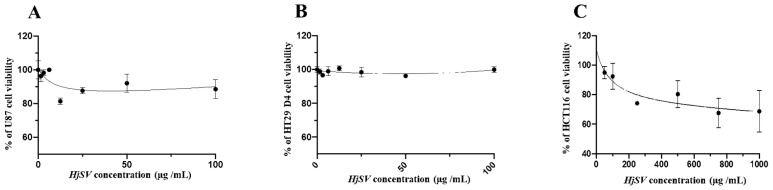
Cytotoxic activity of HjSV on U87 (**A**), HT29 D4 (**B**), and HCT116 (**C**) cancer cell lines. HjSV shows no significant cytotoxic effect on U87 and HT29 D4 cell lines and a non-significant trend toward reduced viability in HCT116 cells at the highest concentration. Data are expressed as mean ± SD (*n* = 3). No statistically significant differences were observed compared to untreated controls (*p* > 0.05). In HCT116 cells, a slight, non-significant decrease in viability (~30%) was noted at the highest tested concentration (1000 µg/mL).

Several studies investigated the in vitro and in vivo anticancer potential of scorpion venoms because of their capacity to promote apoptosis and/or necrosis [33,34]. The cytotoxic activity of HjSV was evaluated on three types of cancer cell lines: U87 (glioblastoma), HT29 D4 (colorectal cancer) and HCT116 (colorectal carcinoma). The results showed that across the tested concentration range (1.55 µg/mL to 100 µg/mL for U87 and HT29 D4, and to 1000 μg/mL for HCT116), cell viability remained high for three cell lines. No statistically significant differences were detected between treated and non-treated cancer cells. This indicates that, under these experimental conditions, HjSV exerts no significant cytotoxic effect on U87 and HT29 D4 cancer cells, and shows a non-significant trend towards reduced viability in HCT116 cells at the very highest tested concentration (Figure 2).

### 2.3. Effects of HjSV on D. melanogaster, A. albopictus and C. pipiens

**Figure 3:**
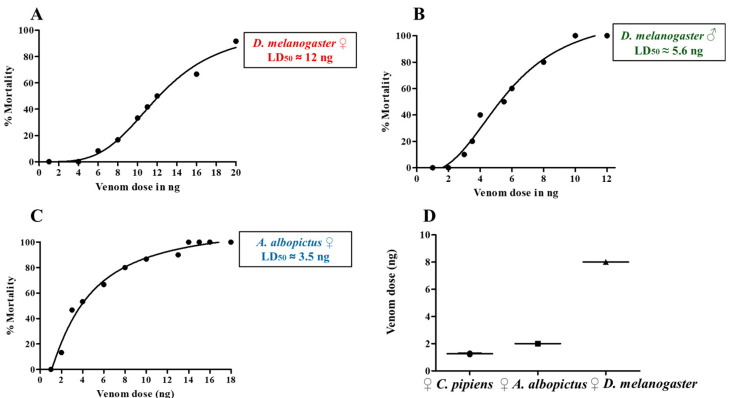
HjSV induces dose-dependent lethality in *D. melanogaster*, *A. albopictus* and *C. pipiens*. Dose–response curves of *D. melanogaster* (females and males) and *A. albopictus* (females) injected with increasing doses of HjSV. The percentage of mortality was plotted against the venom dose (ng). Data points represent observed mortality percentages at each dose (in ng), and the line shows the best-fit nonlinear regression curve (variable slope, 4-parameter logistic model). The calculated median lethal doses (LD_50_) correspond approximately to 12 ng for *D. melanogaster* females (**A**), 5.6 ng for *D. melanogaster* males (**B**) and 3.4 ng for *A. albopictus* females (**C**). Mean minimum venom dose (ng) required to induce mortality in adult females of three different insects (**D**). Each symbol represents the minimum lethal dose determined from an independent experiment (*n* = 3 per species). Horizontal lines indicate the mean ± standard error of the mean (SEM).

To determine the biological effect of HjSV on insects, we first tested its activity on the fruit fly *Drosophila melanogaster*, a powerful genetic model organism suitable for both toxicity and behavior studies. Adult flies were injected with different doses of venom, and survival was monitored in order to establish dose–response relationships and estimate the median lethal dose (LD_50_). In *D. melanogaster* females, mortality was first observed at around 6 ng, with an estimated LD_50_ of 12 ng (Figure 3A). We also evaluated HjSV effect on *D. melanogaster* males, which exhibited greater sensitivity, with mortality observed at doses as low as 3 ng and an LD_50_ of approximately 5.6 ng (Figure 3B). The difference of susceptibility to HjSV between the two sexes is mostly due to body size, as the average weight of females was 1.056 mg and that of males 0.618 mg (Supplementary Table S1). To evaluate the effect of HjSV in other insects, we microinjected two mosquito species that occur in Lebanon (*Aedes albopictus* and *Culex pipiens*) with different venom doses. These mosquitoes differ noticeably from *D. melanogaster* in physiology and ecological niche and are medically important due to their potential role as disease vectors. Results showed that *A. albopictus* females exhibited greater susceptibility than *D. melanogaster*, with mortality occurring at doses as low as 2 ng and an LD_50_ of approximately 3.5 ng (Figure 3C). For *C. pipiens* females’ complete mortality occurred at doses of around 2 ng. Due to this extreme sensitivity, a full dose–response curve was not generated for *C. pipiens*. However, we observed a severe paralytic effect at venom doses of 1 ng (Supplementary Video S1). To illustrate the differences in venom sensitivity between species, we plotted the minimum venom dose that induced observable mortality in each species across three independent experiments. *C. pipiens* exhibited the highest sensitivity, requiring the lowest dose to cause mortality (~1.2 ng). *A. albopictus* required ~2 ng to observe mortality, while *D. melanogaster* was the most resistant, with an approximately four-fold higher minimum lethal dose (Figure 3D). These results prove that HjSV is lethal to the three insects tested with remarkable differences in susceptibility among *Drosophila*, *Aedes* and *Culex*.

### 2.4. Effects of HjSV on Locomotor Activity and Sleep Pattern in D. melanogaster

**Figure 4:**
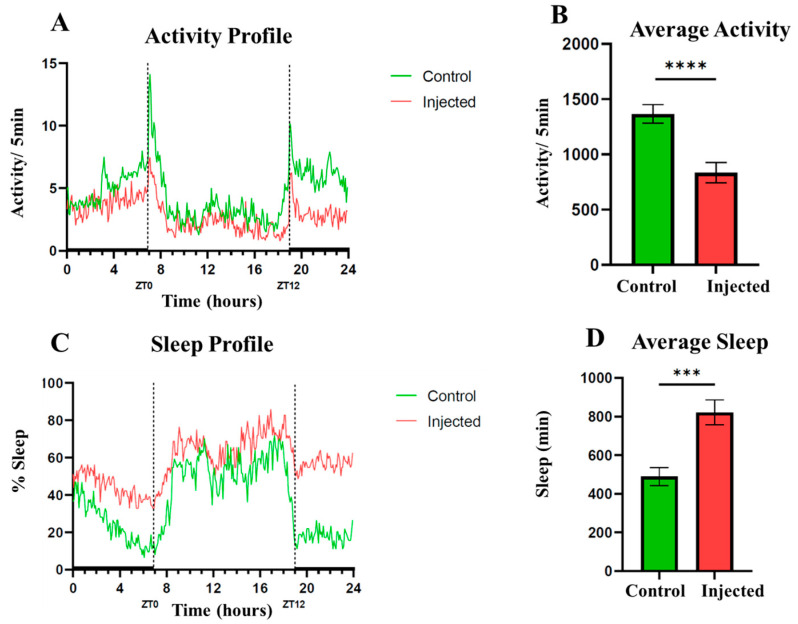
Sub-lethal HjSV exposure alters locomotor activity and increases sleep in females *D. melanogaster*. Female flies injected with 3 ng of HjSV (*n* = 32, red line) were compared to mock-injected flies (*n* = 31, green line). For each group, activity profiles were individually recorded over 2 days then averaged to obtain a representative profile (**A**). The average activity of the flies over 24 h intervals is significantly decreased in the injected flies with respect to the control (**** *p* < 0.0001) (**B**). The sleep pattern of the venom-injected females was also compared to controls. For each group, the percentage of flies sleeping was measured in 5-min intervals and averaged to obtain a representative sleep profile (**C**). The venom-injected flies displayed a significant increase in average sleep time over 24 h period (*** *p* < 0.001) (**D**). Lights were switched off at ZT0 and switched on at ZT12. Activity and sleep averages were obtained using an unpaired *t*-test.

To investigate the neurobiological potential of HjSV apart from its lethal effects, we injected a sublethal dose of venom (3 ng) into *D. melanogaster* females and recorded their activity using the Drosophila Activity Monitor (DAM) system. Under normal light–dark conditions, *D. melanogaster* displays two major peaks of activity: one centered around ZT12 (lights-on, “morning peak”) and another around ZT0 (lights-off, “evening peak”). The data collected showed that HjSV severely affects the flies’ locomotor activity and sleep patterns. Indeed, compared to mock-injected controls, venom-injected flies exhibited a significant reduction in daily locomotor activity (Figure 4A,B). This reduction was consistent throughout the 24 h cycle, including both day and night phases. In addition to reduced activity, venom-injected flies displayed an increase in sleep duration (Figure 4C,D). The sleep profile revealed increased sleep especially during the light phase, suggesting disruption of sleep–wake regulation. These findings confirm that even at doses that do not cause mortality, HjSV alters the behavior of *D. melanogaster*, which is consistent with a neurotoxic effect that impairs both motor and circadian-regulated functions.

## 3. Discussion

Scorpion venoms are a rich reservoir of bioactive molecules, primarily proteins and peptides, with neurotoxins constituting the most abundant and medically important components [35,36]. Neurotoxins act by targeting the prey’s nervous system, specifically disturbing ion channel function. This results in the disruption of nerve signal transmission [37]. While the venom of many Buthidae scorpions has been studied because of its toxicity and therapeutic potential [38,39], the biological activities of HjSV remain poorly explored. In this regard, our study provides functional validation of the recently described proteomic complexity of HjSV [32] by demonstrating its potent and selective bioactivities against insects. While the proteomic analysis revealed an extensive repertoire of ion channel-targeting toxins and enzymes, our work establishes the functional consequences of this molecular diversity in both insect lethality and neurobehavioral modulation.

The protein content analysis of HjSV revealed a complex peptide and protein profile. In fact, the LC-ESI-MS analysis showed that HjSV is mostly dominated by low-molecular-weight molecules in the ~2–7 kDa range, followed by mid- and high-molecular-weight fractions reaching up to ~30 kDa and above. The presence of high molecular weight components may represent large enzymatic proteins. This could be explained by the fact that the venom was obtained through electrical stimulation rather than by manually milking the scorpion’s telson. The most abundant peaks were detected at ~3–5 kDa and ~5–7 kDa, which, according to the literature, most likely correspond to the two major families of scorpion’s neurotoxins: short-chain potassium channel toxins (KTx), and long-chain sodium channel toxins (NaTx), respectively [40,41,42,43]. These results pave the way to the fractionation and identification of the crude venom, which will allow further testing to determine the biologically active components.

The cytotoxicity assays of crude HjSV on three human cancerous cell lines (U87, HT29-D4 and HCT116) revealed no significant effect. This finding contrasts with other studies investigating crude scorpion venom: one study showed that *H. saulcyi* crude venom possesses both in vitro and in vivo cytotoxic effects against breast cancer cells [34]. Another report also showed that *H. Schach* crude venom exhibits in vitro anti-cancer effects on breast cancer cells [44]. The absence of a significant cytotoxic effect in our assays could indicate that HjSV lacks linear cytolytic peptides that have the ability to disrupt mammalian cell membranes. Interestingly, we also showed that HjSV had no antimicrobial activity against a diverse panel of Gram-negative and Gram-positive bacterial strains, even when applied at relatively high doses. This observation is biologically relevant, as antimicrobial and anticancer properties in scorpion venoms are often mediated by shared mechanisms, particularly membrane-disrupting peptides [45].

Consistent with its chemical profile, HjSV showed dose-dependent insecticidal effects in *D. melanogaster* and two mosquito species (*A. albopictus* and *C. pipiens*). This aligns well with the capacity of scorpion venoms to paralyze insects by disrupting voltage-gated sodium and potassium channels [18,40]. Our observation that HjSV causes paralysis then death in mosquitoes corroborates previous findings obtained using blowfly larvae (*Sarcophaga falculata* (*Diptera*)) and cockroaches (*Periplaneta americana*) as models [19]. Indeed, according to Zlotkin et al., HjSV contains both excitatory and depressant toxins that bind to the same sodium channel sites with opposite effects (excitatory toxins cause spastic-contractive paralysis leading to fast death while depressant toxins provoke progressive flaccid paralysis leading to slow death). Interestingly, our findings demonstrated that mosquitoes were more sensitive to HjSV than *D. melanogaster*, despite their larger body size. This observation suggests that susceptibility is not merely a function of dose-per-mass but likely reflects species-specific physiological differences such as detoxification pathways [46] or ion channels variation. In fact, comparative genomic studies showed that mosquitoes have more ion channel gene paralogs than *Drosophila*, potentially providing multiple toxin-sensitive sites [47,48,49]. On another note, the discrepancy between the effects exerted by HjSV on human cancer cells versus insects may be explained by the fact that HjSV targets selective ion channels. In fact, the specificity of HjSV may also explain why *H. judaicus* sting is relatively harmless to humans unlike that of other dangerous Buthidae scorpions [8].

The insecticidal effects HjSV has on medically important vectors, together with the clear variation in susceptibility observed among species (*C. pipiens* being more sensitive than *A. albopictus*, and both mosquitoes being more sensitive than *D. melanogaster*), highlight the potential of HjSV as a novel biopesticide. The species-specific pattern suggests that isolating and characterizing HjSV peptides could yield bioinsecticides with targeted action against particular vector species while minimizing non-target effects. In particular, one study demonstrated that scorpion neurotoxin AaIT from *Androctonus australis* was toxic to insecticide-resistant knockdown (kdr) *D. melanogaster* mutants [50]. This result further supports the idea that scorpion venom peptides can bypass common insecticide resistance mechanisms, possibly by binding to distinct sites on insect sodium channels. Our study revealed that HjSV not only causes lethal paralysis but can also induce profound sublethal behavioral changes in *D. melanogaster*. Indeed, we showed for the first time that flies injected with a sub-lethal dose of HjSV exhibit a significantly reduced locomotor activity and an increased sleep time. This suggests that at low doses, HjSV may act as a neuromodulator, tipping the neural balance towards inhibition rather than overstimulation. As previously mentioned, HjSV possesses depressant toxins that act on sodium channels. Therefore, HjSV may be partially blocking action potential, leading to an immobilized sleep-like condition in insects. The phenotype exerted by HjSV on *D. melanogaster* may reflect what happens in the wild: immobilization of the prey for safe capture.

## 4. Materials and Methods

### 4.1. HjSV Preparation

The venom used was extracted by electrical stimulation and provided by the Lebanese Venom Company (LVC, Karaoun, Lebanon) for exclusive research use and is not registered under a Drug Master File with the Lebanon FDA. Nonetheless, batch-to-batch consistency was ensured through standardized collection, lyophilization, and aliquoting procedures under sterile conditions, and each batch of 1 mg was stored at −20 °C until use. Venom batches were dissolved in phosphate saline buffer prior to their use. For further accuracy, each batch was aliquoted into several tubes, each one containing 1 mL to avoid freezing and thawing of samples.

### 4.2. Liquid Chromatography Electrospray Ionization Mass Spectrometry (LC-ESI-MS) Analysis

LC-ESI-MS analysis of lyophilized venom samples dissolved in 0.01% trifluoroacetic acid (TFA)/H_2_O at a concentration of 2 mg/mL was performed using a Restek Ultra II C18 column (Restek Corporation, Bellefonte, PA, USA) (150 × 2.1 mm, 3 µm particle size). A linear gradient from 0% to 100% buffer B [0.1% (*v*/*v*) TFA/acetonitrile (ACN)] in buffer A [0.1% (*v*/*v*) TFA/H_2_O] was applied to the column at a flow rate of 1 mL/min for 60 min. The column temperature was maintained at 40 °C. Eluted fractions were monitored using a UV detector set at λ = 215 nm.

The venom was injected manually into the LC system and analyzed in positive ion mode using ESI-MS coupled to the LC. ESI spectra were acquired using a Bruker Esquire 3000 ion trap mass spectrometer. The mass spectrometer was calibrated in the m/z range of 50–3000 using the ESI Tuning Mix (Agilent Technologies Deutschland GmbH, Waldbronn, Germany) prior to data acquisition. Source parameters were set as follows: dry gas temperature, 300 °C; dry gas flow, 8 L/min; nebulizer pressure, 30 psi.

Data processing was carried out using Bruker Daltonics software suite (Hystar Version 3.2, Esquire Control Version 1.3, and Data Analysis Version 4.0). For deconvolution and peak picking of the raw spectra, the following parameters were applied in the Data Analysis software: a smoothing width of 3 points (Gaussian algorithm), an S/N threshold set to 3, and the use of the instrument’s “Enhanced” resolution mode. Molecular masses were determined from the charge-deconvoluted spectra.

The LC-ESI-MS raw data files generated in this study are similar and fully consistent with those deposited with the ProteomeXchange consortium via the PRIDE partner repository (identifier PXD063895), corresponding to the raw proteomic data from the same HjSV used in this work [32].

### 4.3. Cell Culture and Cytotoxic Activity Assay

U87, HT29 D4 and HTC116 cancer cell lines were obtained from the American Type Culture Collection (ATCC) and cultured in Dulbecco’s Modified Eagle Medium (DMEM) supplemented with 10% fetal bovine serum (FBS), 100 U/mL penicillin, and 100 µg/mL streptomycin, and maintained at 37 °C in a humidified atmosphere containing 5% CO_2_. The venom cytotoxicity was investigated using the MTT viability test [51]. Briefly, cells were added to a 96-well plate (5000 cells per well) and allowed to adhere for 24 h at 37 °C with 5% CO_2_. Different concentrations of HjSV (ranging from 1.6 µg/mL to 1000 µg/mL) were added to the wells and the plate was incubated for an additional 24 h. After removing the media, a volume of 100 µL of MTT was added to each well. The MTT concentration and the 1-h incubation period were optimized based on preliminary kinetic experiments to ensure linear formazan production without reaching saturation, which can occur with longer incubation times and lead to overestimation of cytotoxicity. This step was performed in the dark, as MTT is photosensitive. The plate was stirred and then incubated at 37 °C for 1 h. The medium was then removed, and 100 µL of DMSO was added to each well to solubilize Formazan crystals. Absorbance was measured at 560 nm with a reference wavelength of 630 nm for background correction. Experiments were run in triplicate. The assay’s sensitivity was validated by a concurrent positive control study where *Jania rubens* extracts showed significant anti-proliferative activity using the identical MTT protocol [52].

### 4.4. Mosquito and D. melanogaster Strains and Rearing

All animal procedures were carried out according to protocols approved by the Institutional Animal Care and Use Committee (IACUC) at the American University of Beirut (AUB), and all methods were carried out in accordance with relevant IACUC guidelines and regulations. Adults were continuously supplied with cotton pads soaked in a 10% sucrose solution and had access to water cups containing clean tap water. Larvae were fed on yeast for the first 24 h, then on fish pellet food until pupation. Pupae were collected with a plastic pipette and placed in water cups inside plastic cages. A local strain of *Aedes albopictus* mosquitoes was maintained in the insectary at 28 °C and 75% humidity using a 12:12 light/dark photocycle. This strain has been kept in the insectary for more than 10 years. Feeding was allowed on anesthetized mice, and eggs were collected on filter paper four days after the blood meal. Eggs were dried for two weeks before inducing hatching by immersion in aged tap water. A local strain of *Culex pipiens* mosquitoes was used in this study. Egg rafts were collected once every generation and were allowed to hatch in tap water. *D. melanogaster W*^1118^ strain was used in all experiments. Flies were reared in 50 mL vials containing standard cornmeal agar food, prepared according to the *D. melanogaster* Bloomington Stock Center recipe. The main stock was kept at 18 °C, with the humidity set at 50%, under a 12:12 light/dark photocycle.

### 4.5. Venom Microinjection and Lethality Assessment

HjSV was injected into the thorax of CO_2_-anesthetized insects using Nanoject III^®^ microinjector (Drummond Scientific, Broomall, PA, USA), at a stock concentration of 1 mg/mL. Injection volumes were adjusted for each individual to achieve the desired dose (ranging from 1.2 to 20 ng/animal). For each dose (ranging from 1 ng to 20 ng), 10–15 insects aged between 5 and 7 days were injected, and equal numbers of controls were injected with water. The number of dead insects was counted after 24 h. A minimum of three independent trials were done for each insect, and one representative graph was shown. Insects that succumbed within one hour following microinjection were disregarded.

### 4.6. Drosophila Activity Assay

The behavior of venom-injected *D. melanogaster* females was recorded using the *Drosophila* activity monitor (DAM) system (TriKinetics Inc., Waltham, MA, USA) and compared to mock-injected flies. Glass activity tubes containing 5% sucrose and 2% agarose, sealed at the food end, and plugged with cotton at the other end were used. Female w1118 flies were loaded into the activity tubes with one fly per tube. The tubes were placed in two monitors, one monitor corresponding to experimental group and the other to the control group. The activity monitors were connected to a data collection system, and the experiment was run for 2 days. The DAM system was maintained at 25 °C and at a cycle of 12 h of daylight and 12 h of dark. Each tube was placed into a channel in which an infrared light beam detects the movement of the fly. Trikinetics data acquisition software (DAMSystem308, Trikinetics Inc.) saves the data as activity of each fly per 5 min. Raw data analysis was carried out using a Microsoft Excel macro considering 5 min of inactivity as sleep and more than 24 h of immobility as death.

### 4.7. Statistical Analysis

Results were expressed as the mean ± standard deviation (SD). All experiments were performed with three independent biological replicates (*n* = 3), with each biological replicate including technical triplicates for cytotoxicity assays. Statistical significance between different samples was analyzed using a two-tailed unpaired *t*-test. Activity and sleep averages were obtained using an unpaired *t*-test. Statistical significance was defined as **** *p* < 0.0001 and *** *p* < 0.001 compared to mock-injected flies. The percentage of mortality at each dose was calculated and plotted against the log-transformed dose. A nonlinear regression analysis with a variable slope (four-parameter logistic model) was used to generate the dose–response curve and estimate the median lethal dose (LD_50_). To compare venom susceptibility across species, the minimum dose at which mortality was first observed was determined for each species in three independent experiments. These values were plotted using dot plots with mean ± standard error of the mean (SEM). All statistical analyses were performed using GraphPad Prism software (Version 10.0.2).

## Data Availability

The original contributions presented in this study are included in the article/Supplementary Material. Further inquiries can be directed to the corresponding author(s).

## Supplementary Materials

The following supporting information can be downloaded at: https://www.mdpi.com/article/10.3390/toxins17110546/s1, Supplementary Table S1: Average body weight of female and male *D. melanogaster*; Supplementary Video S1: Venom-paralyzed *C. pipiens* mosquitoes https://doi.org/10.5281/zenodo.16084545 HjSV Culex pipiens.
