# Supplementary material for: Characterization of Hottentotta judaicus Scorpion Venom: Toxic Effects and Neurobehavioral Modulation in Insect Models
Source: Toxins (Basel). 2025 Nov 3;17(11):546. doi: 10.3390/toxins17110546 (PMC12656134; doi:10.3390/toxins17110546)
Supplement: Supplementary file 1 [file toxins-17-00546-s001.zip › toxins-3862238-supplementary.pdf]

Supplementary Materials: Characterization of *Hottentotta judaicus* Scorpion Venom: Toxic Effects and Neurobehavioral Modulation in Insect Models

Rim Wehbe, Aline Karaki, Zeina Dassouki, Mohamad Rima, Adolfo Borges, Rabih Roufayel, Christian Legros, Ziad Fajloun and Zakaria Kambris

Supplementary Table 1: Average body weight of female and male D. melanogaster.

Sex	Number of Flies Weighed	Total Weight (g)	Average Weight per Fly (mg)
Female	173	0.1827	1.0561
Male	161	0.0996	0.6186
